# Epigenetic regulation in premature ovarian failure: A literature review

**DOI:** 10.3389/fphys.2022.998424

**Published:** 2023-01-04

**Authors:** Jing Wang, Xiguang Sun, Zongxing Yang, Sijie Li, Yufeng Wang, Ruoxue Ren, Ziyue Liu, Dehai Yu

**Affiliations:** ^1^ Department of Reproductive Medicine, Department of Prenatal Diagnosis, Changchun, China; ^2^ Hand Surgery Department, Changchun, China; ^3^ Department of Clinical Laboratory, Changchun, China; ^4^ Department of Breast Surgery, Changchun, China; ^5^ Public Research Platform, The First Hospital of Jilin University, Jilin, China

**Keywords:** premature ovarian failure, epigenetics, DNA methylation, histone modifications, non-coding RNA

## Abstract

Premature ovarian failure (POF), or premature ovarian insufficiency (POI), is a multifactorial and heterogeneous disease characterized by amenorrhea, decreased estrogen levels and increased female gonadotropin levels. The incidence of POF is increasing annually, and POF has become one of the main causes of infertility in women of childbearing age. The etiology and pathogenesis of POF are complex and have not yet been clearly elucidated. In addition to genetic factors, an increasing number of studies have revealed that epigenetic changes play an important role in the occurrence and development of POF. However, we found that very few papers have summarized epigenetic variations in POF, and a systematic analysis of this topic is therefore necessary. In this article, by reviewing and analyzing the most relevant literature in this research field, we expound on the relationship between DNA methylation, histone modification and non-coding RNA expression and the development of POF. We also analyzed how environmental factors affect POF through epigenetic modulation. Additionally, we discuss potential epigenetic biomarkers and epigenetic treatment targets for POF. We anticipate that our paper may provide new therapeutic clues for improving ovarian function and maintaining fertility in POF patients.

## Introduction

Premature ovarian failure (POF) is a reproductive endocrine disease that occurs before the age of 40 in women. POF is the main cause of female infertility and is characterized by increased gonadotropin levels and decreased estrogen levels, accompanied by primary or secondary amenorrhea. POF is highly heterogeneous, and abnormal follicular development at all stages may lead to POF (Collins et al., 2017). Approximately 1% of women under 40% and .1% of women under 30 are estimated to suffer from POF (Coulam et al., 1986; Barbarino-Monnier 2000). POF has become a serious problem that affects the reproductive health of women of reproductive age. The occurrence of POF may be related to various factors, such as an insufficient primordial follicle pool reserve, accelerated follicular atresia, changes in dominant follicle recruitment, and follicular maturation disorders (Webber et al., 2016). The etiology of POF is mostly idiopathic (approximately 80%), and the other causes of POF involve genetic factors, iatrogenic diseases, autoimmune and endocrine diseases, mitochondrial dysfunction, infection and environmental factors (Vujovic 2009). Approximately 20%–25% of POF cases are caused by genetic abnormalities (Qin et al., 2015). The genetic factors conclusively discovered in POF patients to date include autosomal and X chromosome abnormalities and variants in POF candidate genes such as *follicle-stimulating hormone receptor* (*FSHR*), *newborn ovary homeobox gene* (*NOBOX*), *forkhead box L2/O3* (*FOXL2*/*FOX O 3*), *spermatogenesis- and oogenesis-specific basic helix-loop-helix 1* (*SOHLH1*), *folliculogenesis-specific BHLH transcription factor* (*FIGLA*), *growth differentiation factor 9* (*GDF9*) and *bone morphogenetic protein 15* (*BMP15*). In addition to these causative genes, variations in epigenetic and epigenetic regulators also add complexity to the etiology of POF. With the assistance of new technologies represented by next-generation sequencing (NGS), new causative genes, such as non-coding RNAs, have been identified, and researchers have proposed an epigenetic explanation for POF (Jiao et al., 2018).

**FIGURE 1:**
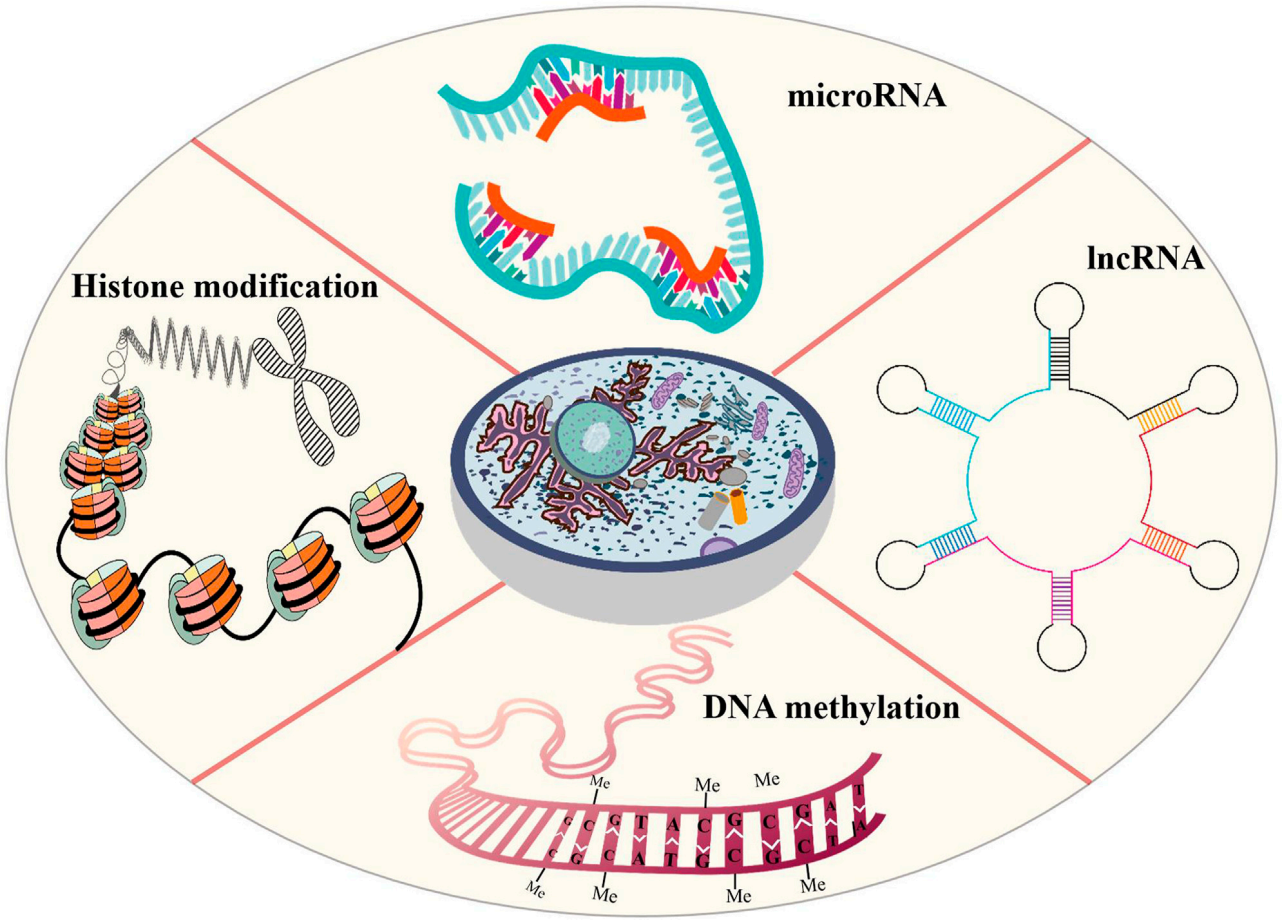
Epigenetic modifications that are probably involved in POI includes DNA methylation, histone modification and non-coding RNA regulation. Altered DNA methylation may lead to gene silencing, reduce gene transcription levels, remodel chromatin and regulate important developmental processes. In POF patients, aberrant DNA methylation may induce GC apoptosis, reduce follicles number and change hormone expression. Histone modifications affect chromatin structure that are conducive to the expression or repression of target genes. Histone modifications may hinder oocyte development and maturation and reduce oocyte number. Non-coding RNAs control gene expression by binding to DNA or RNA sequences and proteins. In POF patients, the abnormal non-coding RNA expression may promote GC apoptosis and promote follicular atresia.

**FIGURE 2:**
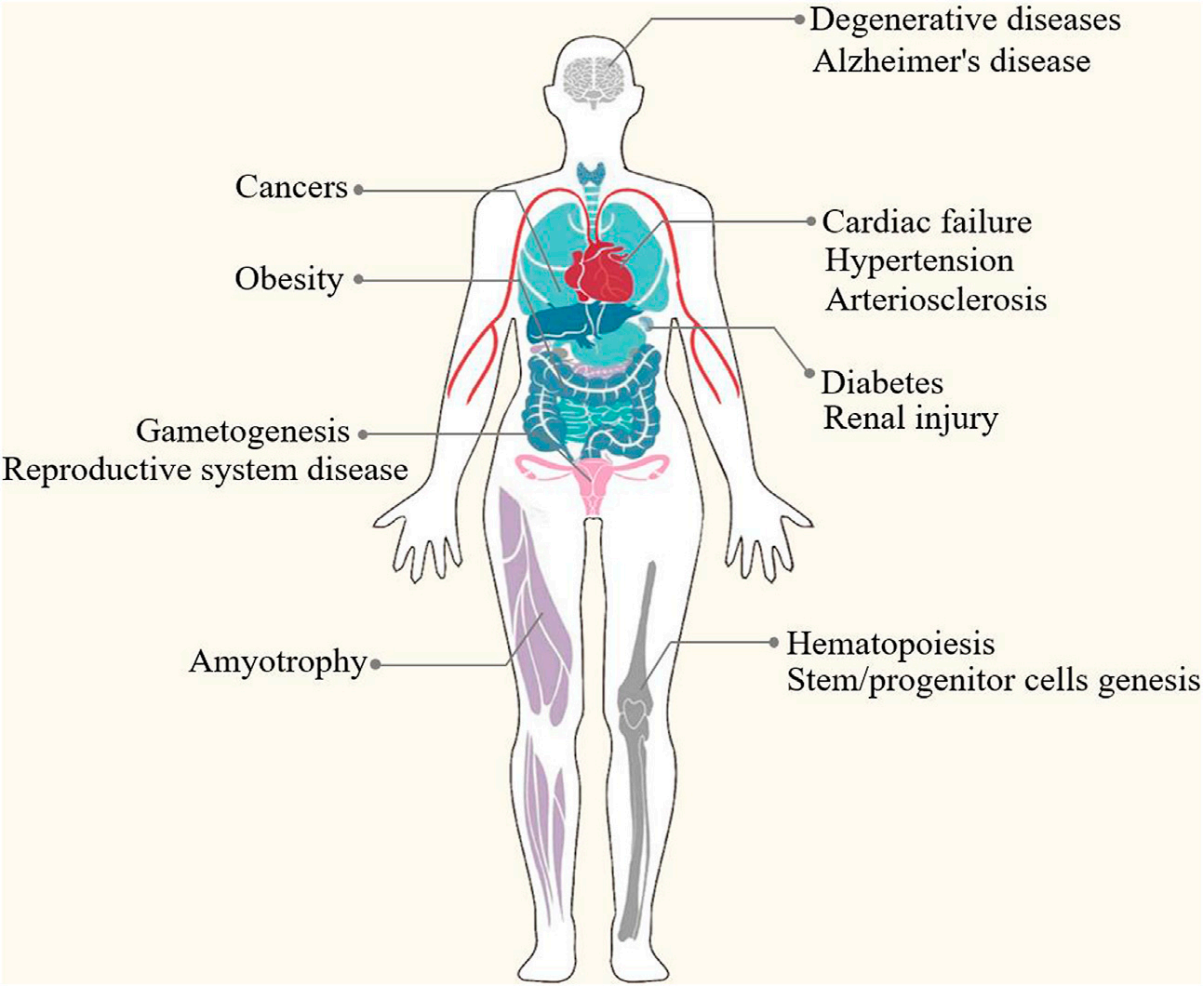
Epigenetics are closely associated with many diseases, such as developmental disorders (e.g., DNA methylation perturbations and loss-of-function mutations of imprinted genes in Beckwith-Wiedemann, Silver-Russell and Fragile X syndromes), complex and multifactorial diseases (e.g., DNA methylation, histone modifications, microRNAs and nucleosome remodeling in cancers, diabetes and obesity) and degenerative disease (e.g., epigenetic modifications in Alzheimer’s disease and amyotrophy).

Epigenetic modification is an indispensable part of cell differentiation, development and activity maintenance (Skvortsova et al., 2018). Epigenetic hallmarks, such as DNA methylation, histone modification and non-coding RNA regulation, may alter chromatin structure without changing the DNA sequence, conferring a differential program of gene expression (Lee et al., 2014) (Figure 1). Since the epigenome of cells is highly plastic and reprogrammable, epigenetic modifications may dynamically and reversibly control gene expression. Epigenetic reprogramming can change cell fate throughout the developmental phase and adulthood, and epigenetic regulation is also an important molecular mechanism by which organisms respond to external environmental factors (Safi-Stibler and Gabory 2020). Epigenetics are closely associated with many diseases, including various developmental disorders, such as Beckwith-Wiedemann, Silver-Russell and Fragile X syndromes (Inoue et al., 2020; Nobile et al., 2021; Tüysüz et al., 2021), and complex and multifactorial diseases, such as cancer, diabetes and obesity (Nebbioso et al., 2018; Ling and Rönn 2019). An increasing numbers of studies has revealed that epigenetic changes are a universal phenomenon in the occurrence and development of POF (Figure 2). In this article, we will provide an overview of the epigenetic mechanism of POF and introduce some potential epigenetic biomarkers and epigenetic treatment targets for POF. Our aim is to provide new therapeutic clues for improving ovarian function and preserving fertility in POF patients.

## Epigenetic mechanisms regulating the occurrence and development OF POF/POI

Epigenetic modifications play a crucial role in reproductive aging (Chamani and Keefe 2019; Li et al., 2021a). Under physiological conditions, epigenetic modification is indispensable in the development of germ cells and early embryos. Many studies have shown that abnormal epigenetic modifications, such as changes in methylation levels, histone modifications and non-coding RNA expression, occur in germ cells in the aging process.

### The effect of DNA methylation on germ cell senescence

DNA methylation is one of the most typical epigenetic modifications and participates in regulating many life activities. Altered DNA methylation is a major epigenetic marker of epigenetic reprogramming during germ cell aging, which leads to gene silencing, reduces gene transcription levels, remodels chromatin, and subsequently regulates important developmental processes, including genomic imprinting and X chromosome inactivation (Das et al., 2009; Deaton and Bird 2011; Duncan et al., 2018; Marshall and Rivera 2018). Aberrant DNA methylation is a classic feature of mammalian aging (Bjornsson et al., 2008; Maegawa et al., 2010). Insufficiency of follicles in the primordial follicle pool is associated with primary POI, and epigenetic modification exerts a strong effect on controlling programmed oocyte death during the establishment of the primordial follicle pool (PF) (Sun et al., 2017; Sun et al., 2018). Liu et al. found that the methylation levels of GATCG sites in oocytes decreased from primary follicles to secondary follicles, while the methylation levels of CCGG and GATCG in ovarian granulosa cells (GCs) decreased significantly from primary to secondary follicles and then increased in tertiary follicles. Moreover, the authors observed marked demethylation of CCGG sites in terminal deoxynucleotidyl transferase dUTP nick-end labeling (TUNEL)-positive GCs, suggesting that failure of the follicular stage-dependent increase in CCGG and GATCG methylation may induce GC apoptosis and follicular atresia (Liu et al., 2018b). In addition, Yu et al. conducted mRNA-Seq and genome-wide DNA methylation studies in human ovarian GCs and found that compared with young healthy donors, older women with a natural age-related decline in ovarian function showed lower gene expression (1,809 genes were downregulated) that was correlated with higher gene body methylation and 3′-end GC density (Yu et al., 2015). According to Marshall et al., the expression level of the DNA methyltransferase Dnmt1 in oocytes increased in 69- to 70-week-old mice compared with 10- to 13-week-old mice, and the DNA and histone H3K9me2 methylation levels also increased (Marshall et al., 2018). However, Yue et al. found that the DNA methylation level of metaphase II (MII) oocytes in 35- to 40-week-old mice decreased compared with six- to 8 week-old mice, and the expression levels of Dnmt1, Dnmt3a, Dnmt3b, and Dnmt3L decreased (Yue et al., 2012).

Several genes associated with ovarian function are regulated by epigenetics, and their epigenetic variations may lead to POF. Anti-Mullerian hormone (AMH), a GC-rich gene, is a biomarker for diminished ovarian reserve (Moolhuijsen and Visser 2020). As shown in the study by Yu et al., AMH expression was strikingly downregulated in the poor responder group, and a partially methylated CpG island was identified close to its transcriptional end site (TES) (Yu et al., 2015). Gao et al. examined pigs and found that excess sodium fluoride (NaF) increased the DNA methylation level and downregulated the expression of the maternal imprinted gene NNAT (neuronatin), which affects glucose transport by inhibiting the phosphoinositide 3-kinase (PI3K)- AKT serine/threonine kinase 2 (Akt2) signaling pathway. Suppression of NNAT disrupts glucose metabolism in oocytes, hinders oocyte maturation, affects follicular development and reduces ovarian reserve (Gao et al., 2016; Liu et al., 2018c). Kordowitzki et al. studied the DNA methylation-based biomarkers of aging (epigenetic clocks) in bovine oocytes and blood to reveal the epigenetic mechanisms underlying the effects of aging on the female reproductive system, and they found that the rate of epigenetic aging was slower in oocytes than in blood, but oocytes appeared to begin aging at an older epigenetic age. Their findings suggested that epigenetic clocks for oocytes are promising markers to address questions of reproductive aging, including methods to slow the aging of oocytes (Kordowitzki et al., 2021). Guglielmino et al. observed significantly lower expression of TAp73 (encoded by the *P73* gene, which belongs to the *P53* family) in the oocytes of women over 38 years old than in women under 36 years old; they also indicated that the methylation level of CpG sites in the *P73* promoter decreased in aging mouse oocytes, indicating that CpG hypomethylation may be involved in oocyte senescence (Guglielmino et al., 2011). Qian et al. measured the expression levels of 5-cytosine (5 mC) and ten-eleven-translocation (Tet)/thymine DNA glycosylase (Tdg) in mouse oocytes obtained under natural and accelerated aging conditions, and they found that the levels of intermediates produced by demethylation-modified cytosine (5mC, 5 hmC, 5 fC and 5caC) increased. Additionally, Tet expression levels increased and Tdg expression levels decreased. Notably, genomic DNA demethylation was more significant in chemically induced senescent oocytes (Qian et al., 2015).

DNA methylation also occurs at sites other than CpG, known as non-CpG methylation or asymmetric methylation. Yu et al. showed that human genomic CpG methylation is mostly stable during oocyte maturation, but non-CpG methylation increases in local genomic regions and gradually accumulates (Yu et al., 2017). This finding is consistent with conclusions drawn by Tomizawa, who reported that the methylation of non-CpG sites changes dynamically during the maturation of mouse oocytes (Tomizawa et al., 2011).

Female X -chromosome inactivation (XCI) is an important epigenetic mark and leads to differences in epigenetic marks on the active and inactive X -chromosome (Boumil and Lee 2001). The CpG island-containing promoters of genes subject to XCI are approximately 50% methylated in females (Cotton et al., 2011). XCI is a random process, and the number of cells expressing maternal and paternal chromosomes is 50:50. When XCI is not random, an imbalance of cells expressing either the paternal or maternal X–chromosome occurs, which is known as skewed X -chromosome inactivation (sXCI) (Liehr et al., 2018). Studies have suggested that POF correlates with sXCI. For example, Susana et al. suggested that sXCI and X -chromosome deletion may induce abnormal expression of some crucial ovarian development-associated genes and induce POF (Ferreira et al., 2010). Sato et al. also implied that sXCI disrupts the expression of genes involved in ovarian development (such as bone morphogenetic protein 15 (BMP15), a member of the transforming growth factor-β (TGF-β) superfamily that regulates follicular survival/atresia and oocyte maturation), which in turn causes ovarian dysfunction and subsequently results in POF (Sato et al., 2004). sXCI is commonly observed in diploid cell lines arising from trisomy rescue events (Peñaherrera et al., 2000). Oocytes may contain high levels of trisomic cells, and these cells are more likely to suffer early atresia, resulting in a reduced follicular pool size and the occurrence of primary amenorrhea (Bretherick et al., 2007). However, Silvia et al. analyzed 151 POF patients and found that the distribution of sXCI in the POF population was similar to that in the control population. They also suggested that small deletions or mutations in X-linked genes do not appear to be a common feature of POF patients and that X-linked genes involved in POF may be too few or are unable to interfere with XCI (Bione et al., 2006). Marian et al., Pu et al. and Sang et al. also reported that sXCI may not be associated with POF (Kline et al., 2006; Yoon et al., 2008; Pu et al., 2010; Spath et al., 2010).

DNA methylation, one of the most classic epigenetic regulation patterns, affects a variety of cellular behaviors by regulating gene transcription. Numerous studies have widely confirmed that DNA methylation contributes to various diseases, particularly degenerative diseases. However, all the literature we can find is focused on DNA methylation of germ cells (Table 1), and the large-scale detection of DNA methylation in the occurrence and development of POF and in-depth systematic functional research of POF have not been conducted.

**TABLE 1:** Effects of DNA methylation on germ cells.

Genes	Methylation level	Effects on germ cells	References
*AMH*	Rise	Promoting GC senescence and apoptosis	Yu et al. (2015)
*NNAT*	Rise	Promoting oocyte senescence	Liu et al. (2018c)
*Dnmt1,Dnmt3a,Dnmt3b,Dnmt3L*	Rise	Being involved in oocyte aging process	Marshall et al. (2018)
*DENND1A*	Rise	Being involved in egg cell aging process	Kordowitzki et al. (2021)
*TAp73, P73*	Decline	Being involved in oocyte aging process	Guglielmino et al. (2011)

### The effect of histone modifications on germ cell senescence

Histone modifications are covalent posttranslational epigenetic modifications that alter the chromatin structure and subsequently regulate gene expression. Histones are responsible for packing DNA into small structures called nucleosomes within the nucleus. Histones are composed of octamers with two copies each of H2A, H2B, H3 and H4 encapsulating DNA and a linker histone (H1). Chemical modifications of histone tails, such as acetylation, methylation, phosphorylation and ubiquitination, alter the affinity of chromatin for those transcription factors, thereby affecting gene transcription and cellular phenotypes (Barth and Imhof 2010). Histone acetylation is a switch that allows interconversion between permissive and repressive chromatin domains (Verdone et al., 2005); histone methylation contributes to the regulation of genome integrity, replication and accessibility (Li et al., 2021b); histone phosphorylation and ubiquitination facilitate DNA damage repair and maintain genome stability (Mattiroli and Penengo 2021; Zhu et al., 2021); and errors in histone posttranslational marks have been implicated in the pathogenesis of human disorders (Eiras et al., 2022). Although direct evidence that histone modifications are associated with POI/POF is unavailable, we can obtain some proof showing how histone modifications regulate mammalian oocyte meiosis, growth, maturation and activation during the normal aging process (Swain et al., 2007; Gu et al., 2010; Yang et al., 2012a). For example, Bui et al. investigated the changes in histone H3 in pig oocytes and found that it undergoes the acetylation/deacetylation and phosphorylation/dephosphorylation modifications during the growth, maturation, and activation of pig oocytes. They also noted that phosphorylation of histone H3 is a key event in oocyte meiosis (Bui et al., 2007). The decreases in oocyte competence with maternal aging is a major factor contributing to mammalian infertility, and one of the factors contributing to this infertility is changes in chromatin modifications. Shao et al. compared H3K4 methylation (H3K4me1/2/3) in young (6–8 weeks old) and older (42–44 weeks old) mouse oocytes and found that H3K4me2/3 levels decreased in older germinal vesicles (GVs), while H3K4me2 levels subsequently increased in older MII oocytes. The authors also found that the expression level of *Kdm1a* (the gene encoding the H3K4 demethylase lysine (K)-specific demethylase 1A) was increased in older GV oocytes but decreased in older MII oocytes, which was negatively correlated with H3K4me2 levels. These epigenetic changes represent a molecular mechanism underlying human infertility caused by aging (Shao et al., 2015). Suo et al. studied acetylated H4K12 (acH4K12) levels in oocytes during mouse aging and evaluated their effect on the developmental potential of oocytes. They stated that the acH4K12 levels in oocytes were significantly increased during mouse aging. When histone acetylation of oocytes was artificially increased by trichostatin A (TSA) treatment in young mice, a large number of oocytes failed to form pronuclei or formed morphologically abnormal pronuclei (Suo et al., 2010). Manosalva et al. reported that acH4K12 and acH4K16 levels decreased in old GV oocytes, while acH4K12 levels subsequently increased in old MII oocytes. Additionally, the expression of cell division cycle gene 2a (Cdc2a, a gene related to H4K12 acetylation) increased in old oocytes with a non-surrounded nucleolus but decreased in old MII oocytes. Correction of the histone deacetylation of old oocytes at the GV stage restores young-like levels of H4K12 acetylation and CDC2A protein at the MII stage. These data provide evidence for the mechanism by which histone modification affects aging-induced infertility (Manosalva and González 2009). Similar conclusions were reported in the studies by Akiyama et al., Zhang et al., van den Berg et al. and Eslami et al., who suggested that histone modification may affect the aging of germ cells (Akiyama et al., 2006; Eslami et al., 2018; van den Berg et al., 2011; Zhang et al., 2014).

Histone modifications mediate a variety of critical biological processes through chromatin modifications that are conducive to the expression or repression of target genes. Table 2 summarizes the effects of histone modifications on germ cells. Unfortunately, few studies have directly addressed the association of histone modifications with POF. Additionally, the bulk of the literature describing ovarian function has focused on the acetylation and methylation of histones, but other modifications, such as phosphorylation, ubiquitination, and lactylation, have received little attention, although they are also crucial histone epigenetic modifications.

**TABLE 2:** Effects of histone modifications on germ cells.

Genes	Expression level	Histone modification	Effects on germ cells	References
*Cbx1*	Increase	Decreased methylation levels of H3K36me2/H3K79me2/H4K20me2	Interfering oocyte growth and maturation	Manosalva and González (2010)
*Sirt1*	Decrease	Decreased methylation levels of H3K36me2/H3K79me2/H4K20me2	Hindering oocyte development and maturation	Manosalva and González (2010)
*Kdm1a*	Decrease/Increase	Elevated methylation levels of H3K4me2/Decreased methylation levels of 3K4me2 and me3	Being involved in MII oocyte egg senescence/GV oocyte aging process	Shao et al. (2015)
*BRCA1*	Decrease	Decreased methylation and acetylation levels of H3K9	Hindering oocyte maturation	Pan et al. (2008)
*Cdc2a*	Decrease	Increased acetylation level of H4K12/Decreased acetylation level of H4K12 and H4K16	Being involved in MII/GV oocyte senescence process	Manosalva and González (2009)
*Sirt2*	Decrease	Elevated acetylation level of H4K16	Affecting oocyte quality	Zhang et al. (2014)
*FMR1*	Decrease	Increased methylation levels H3K9ac and H3K9me	Reducing the number of oocytes	Eslami et al. (2018)

### Epigenetic enzymes involved in germ cell senescence

Epigenetic-related enzymes directly regulate epigenetic-based gene expression and therefore exert considerable effects on the aging process and age-associated diseases. DNA methyltransferases are a class of crucial epigenetic enzymes, and include DNMT1, DNMT2, DNMT3A, DNMT3B, DNMT3C and DNMT3L. DNMT1 maintains the methylation of DNA that has been established in the genome, while DNMT3a and DNMT3b are essential for *de novo* methylation (Finnegan and Kovac 2000). Numerous studies have confirmed the role of DNMT enzymes in transcriptional silencing through their ability to methylate gene promoters and change chromatin states (Klose and Bird 2006; Lyko 2018). However, recent studies found that under certain circumstances, DNMTs may cooperate with transcription factors to activate gene transcription (Rinaldi et al., 2016; Yin et al., 2017). DNA methyltransferases are essential for mammalian development and have been proven to exhibit remarkable differences in spatial and temporal expressional levels and subcellular localizations (Petrussa et al., 2014; Uysal et al., 2017; Chen and Zhang 2020). Yu et al. measured the expression of DNA methyltransferases (Dnmt1/3a/3b/3L) in MII oocytes and found that Dnmt protein levels in the old group were lower than those in the young group, and the DNA methylation levels were also decreased significantly during mouse aging, suggesting that the decreased expression of DNA methyltransferases and the change in genome-wide DNA methylation in oocytes may be related to lower reproductive potential in old female mice (Yue et al., 2012).

TET enzymes include TET1, TET2 and TET3, which regulate DNA demethylation and transcription (Dai et al., 2016; Wu and Zhang 2017). TET-mediated DNA demethylation occurs in various biological contexts, including primordial germ cell development, somatic cell reprogramming, embryonic stem cell maintenance and tumorigenesis (Wu and Zhang 2017). Studies have also revealed the involvement of TET and active DNA demethylation in genomic instability and DNA damage repair (An et al., 2015; Georges et al., 2022). TET1 is necessary for maintaining oocyte quality, oocyte number and the follicle reserve. Tet1 deficiency promotes DNA demethylation in primordial germ cells, leading to downregulation of meiotic gene expression, which in turn leads to abnormal ovarian cell meiosis and a reduced follicle reserve (Yamaguchi et al., 2012). Liu et al. also found that mouse Tet1 deficiency downregulates the expression of X -chromosome-linked genes, such as *fragile X messenger ribonucleoprotein 1* (*Fmr1*), substantially reducing the follicle reserve of *Tet1*-deficient mice at a young age, and the follicle reserve further decreases with age, a phenomenon consistent with POF (Liu et al., 2021a). Wang et al. showed that Tet2 deficiency increases the DNA methylation levels of genes involved in oocyte meiosis, such as *meiotic double-stranded break formation protein 1* (*Mei1*), *cyclin B3* (*Ccnb3*), *meiosis specific with coiled-coil domain* (*Meioc*), synaptonemal complex protein 1/2 (*Sycp1*/*Sycp2*), *mutS homolog 5* (*Msh5*), *RAD21 cohesin complex component like 1* (*Rad21l*) and *PR domain containing 9* (*Prdm9*), which significantly delays meiotic progression, reduces oocyte quality and mouse fertility, and thereby accelerates reproductive aging in adult female mice (Wang et al., 2021a).

Primordial follicles (PFs) are the initial stage of follicle development, and their number determines the length of the female reproductive lifespan (Adhikari and Liu 2009). The balance between the quiescent and activated states of PFs is crucial to female fertility, and thus excessive activation may deplete quiescent follicle reserves (Reddy et al., 2010). Histone deacetylases (HDACs) include HDAC1, HDAC2, HDAC3, SIRT1, HDAC6 and the lysine-specific demethylase 1 (LSD1). Tighter wrapping of DNA around histones diminishes accessibility for transcription factors and leads to transcriptional repression. HDAC enzymes remove the acetyl group from histones, resulting in a decrease in the space between the nucleosome and the DNA (de Ruijter et al., 2003). HDACs regulate PF maintenance, oocyte maturation, ovulation and early embryonic development (Vaquero et al., 2007; Matoba et al., 2014; Ma and Schultz 2016; Wang et al., 2019; He et al., 2020). HDAC6 is expressed at high levels in germ cells and is involved in maintaining primordial follicle dormancy in neonatal mice (Verdel et al., 2000; Kawaguchi et al., 2003; Bertos et al., 2004; Zhang et al., 2007; Aslan et al., 2013; Li et al., 2013). Zhang et al. found that overexpression of HDAC6 in mouse embryonic stem cells may reduce the level of H3K9me3, increase follicle numbers (especially antral and secondary follicles) and prolong the reproductive lifespan of mice (Zhang et al., 2017). Zhang et al. support these conclusions and stated that HDAC6 may maintain mouse primordial follicles in a dormant state by regulating the mechanistic target of rapamycin (mTOR)- KIT ligand (KITL) signaling pathway (Zhang et al., 2021). Conversely, low expression of Hdac6 may diminish ovarian reserve. Sirtuin 1 (Sirt1, an NAD-dependent deacetylase) is upregulated during mouse PF activation and activates the Akt/mTOR pathway, suggesting that Sirt1 participates in maintaining PF quiescence (Zhang et al., 2019c). According to He et al., specific disruption of Lsd1 resulted in significantly increased autophagy through its H3K4me2 demethylase activity and a decreased oocyte number in perinatal mice, leading to the depletion of oocytes (He et al., 2020). In contrast, some studies confirm that treatment with HDAC inhibitors, such as TSA or butyrate, may promote steroid hormone synthesis in GCs during follicular development by differentially regulating gene expression (Xing et al., 2018; Ye et al., 2021).

Epigenetic enzymes recognize, add and remove epigenetic marks on DNA and histones. Changes in the structure and activity of epigenetic enzymes significantly affect life expectancy and are associated with aging-related structural and functional declines and senile diseases (Prachayasittikul et al., 2017). Over the past few years, epigenetic drugs, as represented by inhibitors of DNMTs and HDACs, have emerged and have been proven to potentially affect aging-associated diseases and longevity (Pasyukova et al., 2021). Unfortunately, none of these DNMT inhibitors have been proven to prevent POF, and some of them may even impair ovarian function (Table 3). Therefore, relevant research and market development are urgently needed and promising.

**TABLE 3:** Effects of epigenetic enzymes on germ cells.

Enzymes	Variation or function of enzymes	The effect of enzymes on germ cells	References
Dnmt	Dnmt1/Dnmt3a/Dnmt3b/Dnmt3L hypomethylation	Promoting oocyte senescence	Yue et al. (2012)
Tet1	Demethylating and downregulating the meiosis-related genes such as FMR1	Decreasing the number of oocytes and reducing ovarian reserve	Liu et al. (2021a)
Tet2	Elevating DNA methylation and downregulating the meiosis-related genes in oocytes	Decreasing oocyte quality	Wang et al. (2021a)
HDAC6	Increasing the acetylation level of H3K9me3/H3K9ac/H4K8, upregulating the expression level of Bax, and downregulating the expression level of GLUT3/GLUT8/Bcl-xl	Depleting primordial follicles, reducing ovarian reserve, promoting GCs apoptosis, and inhibiting production of reproductive hormones such as estrogen	Zhang et al. (2017)
LSD1	Demethylating H3K4me2 and interfering transcription of p62	Increasing autophagy level in oocyte, promoting oocyte depletion and reducing ovarian reserve	He et al. (2020)

### Non-coding RNAs involved in the occurrence and development of POF

Non-coding RNAs (ncRNAs) are epigenetic marks that control gene expression by binding to DNA or RNA sequences (at the transcriptional level) and proteins (at the posttranscriptional level). The main types of ncRNAs are microRNAs (miRNAs, ∼20 nt in length) and long ncRNAs (lncRNAs, >200 nt in length), which are involved in multiple physiological and pathological processes (Esteller 2011; Lee and Young 2013).

#### miRNAs and POF

MiRNAs play a pivotal role in mammalian follicular cell physiology, ovarian function and oocyte maturation by regulating the expression of fertility-related genes (Tesfaye et al., 2018). Studies have suggested that miRNAs are associated with multiple reproductive diseases, such as infertility, polycystic ovary syndrome (PCOS), POF and repeated implantation failure (RIF) (Mezzanzanica et al., 2011; Kamalidehghan et al., 2020). However, the underlying mechanisms remain unclear.

Many researchers have focused on how miRNAs modulate POF occurrence and development. The most popular topic is how miRNAs affect ovarian GCs. Follicular atresia removes most follicles from the ovaries before ovulation and thus limits mammalian follicle utilization. However, GC apoptosis is the basic physical mechanism of follicular atresia. Currently, the broad consensus is that miRNAs participate in the regulation of GC apoptosis. Zhang et al. compared the miRNA profiles of different mammalian species and found that the let-7 family, miR-23–27–24 cluster, miR-183–96–182 cluster, miR-17–92 cluster and their related pathways are involved in GC apoptosis and follicular atresia (Zhang et al., 2019b). Liu et al. observed the downregulation of miR-92a expression in atretic porcine follicles, and miR-92a expression inhibited GC apoptosis by targeting the Smad family member 7 (Smad7)-TGFβ pathway (Liu et al., 2014). Yang et al. analyzed the miRNA-regulated signaling pathways and related genes in POF patients by performing Gene Ontology (GO) and Kyoto Encyclopedia of Genes and Genomes (KEGG) pathway analyses and found that miR-23a was overexpressed in POF patients. They reported that miR-23a may induce GC apoptosis by activating the X-linked inhibitor of apoptosis protein (XIAP) and caspase-3 pathways (Yang et al., 2012b). Chen et al. observed the significant upregulation of miR-146a expression in GC cells from POF patients, and miR-146a induced GC apoptosis by targeting IRAK1 (interleukin-1 receptor-associated kinase) and TRAF6 (tumor necrosis factor receptor-associated factor 6) (Chen et al., 2015). Zhang et al. reported significantly higher miR-181a expression in blood from POF patients. They also suggested that overexpression of miR-181a downregulates cyclin D2 and inhibits mouse GC proliferation (Zhang et al., 2013). According to Zhang et al. and Dang et al., the expression of miR-127-5p and miR-379-5p in biochemical POI patients was significantly upregulated. Overexpression of these two miRNAs may inhibit mouse GC proliferation and attenuate DNA repair by targeting high mobility group box 2 (Hmgb2), poly(ADP-ribose) polymerase 1 (Parp1) and X-ray repair cross complementing 6 (Xrcc6) (Dang et al., 2018; Zhang et al., 2020). Other similar studies investigating the correlation between miRNA variations, gene regulation, GC apoptosis, folliculogenesis and POF development are listed in Table 4.

**TABLE 4:** MiRNAs involved in POF.

miRNAs	Expression level	Target genes	Regulation results	References
miR-29a	Decrease	*PLA2G4A*	Promoting follicular atresia	Kuang et al. (2014)
miR-144	Decrease	*mTOR*	Promoting follicular atresia	Kuang et al. (2014)
miR-27 b	Increase	*PappA*	Inducing ovarian GC apoptosis	Kamalidehghan et al. (2020)
miR-190	Increase	*PHLPP*	Activating primordial follicle pool and reducing ovarian reserve	Kamalidehghan et al. (2020)
miR-151	Increase	*TNFSF10*	Inducing ovarian GC apoptosis	Kamalidehghan et al. (2020)
miR-672	Increase	*FNDC1*	Inducing ovarian GC apoptosis	Kamalidehghan et al. (2020)
miR-1275	Decrease	*CYP19A1/LRH-1*	Promoting early apoptosis of porcine GCs and initiating follicular atresia; Inhibiting E2 release	Liu et al. (2018a)
miR-361-5p	Decrease	*VEGFA*	Promoting porcine GCs apoptosis	Ma et al. (2020)
miR-26 b	Decrease	*HAS2*	Enhancing ovarian GC apoptosis	Liu et al. (2016)
miR-22	Decrease	*SIRT1*	Suppressing GC apoptosis	Xiong et al. (2016)
miR-92a	Decrease	*Smad7*	Inducing ovarian GC apoptosis	Liu et al. (2014)
miR-181a	Decrease/Increase	*S1PR1/acvr2a*	Promoting follicular GC apoptosis/Suppressing mouse GC proliferation	Zhang et al. (2019a); Zhang et al. (2013)
miR-23a	Increase	*XIAP/Caspase-3*	Promoting human GC apoptosis	Liu et al. (2019a); Yang et al. (2012b)
miR-27a	Increase	*Caspase/SMAD5*	Promoting human GC apoptosis	Liu et al. (2019a)
miR-379-5p	Increase	*Parp1/Xrcc6*	Inhibiting GC proliferation and attenuating DNA repair efficiency	Dang et al. (2018)
miR-127-5p	Increase	*Hmgb2*	Impairing GCs function	Zhang et al. (2020)
miR-146a	Increase	*IRAK1/TRAF6*	Promoting ovarian GC apoptosis	Chen et al. (2015)
miR-155	Decrease	*MSH2*	Downregulating cell cycle and DNA replication related genes in theca cells	Donadeu et al. (2017)
miR-378	Decrease	*VEGFA*	Regulating steroidogenesis in GCs and promoting follicular atresia	Donadeu et al. (2017)
miR-222	Decrease	*ETS1*	Promoting angiogenesis in theca cells	Donadeu et al. (2017)
miR-199a-5p	Decrease	*HIF1A*	Inducing follicular GC apoptosis	Donadeu et al. (2017)
let-7g	Decrease	*TGFBR1*	Inducing GC apoptosis and follicular atresia	Zhou et al. (2015)
miR-22-3p	Decrease	*FSH*	Inhibiting FSH levels and reducing estradiol synthesis	Dang et al. (2015)
miR-21	Increase/Decrease	*SNHG7/Peli1*	Inhibiting ovarian GC proliferation/Regulating the proportion of Tregs and destroying ovarian tissue	Aldakheel et al. (2021); Li et al. (2020b)
miR-146aC>G	Decrease	*FOX O 3/FOXL2/C*C*ND2*	Interfering follicle development	Cho et al. (2017)
miR-196a2T>C	Decrease	*DICER1, FAS, NOBOX*	Interfering folliculogenesis	Rah et al. (2013)
miR-15 b	Increase	*α-Klotho Kl*	Inducing ovarian GC apoptosis	Liu et al. (2019b)
miR-125a-5p	Increase	*Stat3*	Inducing ovarian GC apoptosis	Wang et al. (2016)
miR-15a	Increase	*Last1*	Inducing ovarian GC cytotoxicity, senescence and apoptosis	Ai et al. (2018)

The use of drugs is a negligible risk factor for POF. A clear understanding of the molecular mechanism by which these drugs induce POF is important for protecting the female reproductive system in women of childbearing age (especially female cancer patients). Multiple studies have revealed that miRNAs are involved in drug-induced POF. For example, Liu et al. found that cyclophosphamide may upregulate the expression of miR-15b, silence endogenous α-Klotho (KL) and stimulate the TGFβ1/Smad pathway, attenuating the autophagy of mouse GCs, inducing GC apoptosis and reducing ovarian reserve (Liu et al., 2019b). Wang et al. reported that cisplatin may upregulate miR-125a-5p expression and induce mouse GC apoptosis by inhibiting the expression of signal transducer and activator of transcription 3 (Stat3) (Wang et al., 2016). As shown in the study by Ai et al., triptolide induces the expression of endogenous miR-15a and inhibits the Hippo-yes-associated protein (Hippo-YAP)/TAZ pathway, leading to the cytotoxicity, senescence and apoptosis of ovarian GCs (Ai et al., 2018). Additionally, epigenetic therapy is a promising approach for the treatment of cancers (Matthews et al., 2021; Xu et al., 2021; Brown et al., 2022; Hoang and Landi 2022). Since DNA methylation plays an important role in the formation of primordial germ cells, epigenetic therapy, e.g., DNMT inhibitor-based (such as decitabine) therapies, may hamper oocyte differentiation and development and thus induce POI (Yang et al., 2019). However, studies and experimental results in this area are still lacking and needed.

#### LncRNAs and POF

LncRNAs function in various diseases (Yang et al., 2014). Studies have found that lncRNAs may participate in the development of POF, but the underlying mechanism remains unclear.

Two mechanisms may explain the role of lncRNAs in POF. One is the interactions between lncRNAs and miRNAs, which may induce ovarian GC apoptosis. For example, Zheng et al. found that in POF patients, the lncRNA deleted in lymphocytic leukemia 1 (DLEU1) is upregulated and its overexpression promotes GC apoptosis (Zheng et al., 2021). Zhang et al. observed a decreased expression level of translation regulatory long non-coding RNA 1 (TRERNA1) in POF patients, and TRERNA1 may sponge miR-23a and suppress GC apoptosis (Zhang et al., 2022). Yu et al. found that the lncRNA BBOX1 antisense RNA 1 (BBOX1-AS1) in GC was unregulated in POF patients. BBOX1-AS1 may directly interact with miR-146b, and overexpression of BBOX1-AS1 may increase GC apoptosis in POF by sponging miR-146 b (Yu et al., 2022). Yao et al. documented that non-coding RNA that was highly expressed in atretic follicles (NORHA), which is related to follicular atresia, induces GC apoptosis by inhibiting the activities of the miR-183–96–182 cluster and FoxO1 axis (Yao et al., 2021).

The other mechanism is that some lncRNAs are involved in regulating several critical proteins and signaling pathways. For example, Xiong et al. indicated that cyclophosphamide induces mouse ovarian atrophy and inhibits the proliferation of ovarian GCs. The lncRNA maternally expressed gene 3 (Meg3) was unregulated in cyclophosphamide-treated mouse ovarian GCs, and inhibition of Meg3 effectively reduced the effect of cyclophosphamide through the p53-p66Shc pathway (Xiong et al., 2017). Li et al. observed the significant downregulation of the expression of the lncRNA NEAT1 (nuclear enriched abundant transcript 1) in the ovarian tissue of POF patients. Cytological experiments indicated that overexpression of NEAT1 may inhibit ovarian cell apoptosis by inhibiting P53 (Li et al., 2020a). Zhao et al. detected significantly lower expression of the lncRNA HOX antisense intergenic RNA (HOTAIR) in ovarian tissue and serum samples from POF patients than in healthy controls. Overexpression of HOTAIR in hamster ovary cell lines upregulated Notch-1 expression and reduced cell apoptosis (Zhao and Dong 2018). Table 5 summarizes the lncRNAs involved in POF and their targets.

**TABLE 5:** POF-related lncRNAs and their targets.

lncRNA name	Expression level	Targets	Regulation results	References
NEAT1	Decrease	*P53*	Inducing ovarian GC apoptosis	Li et al. (2020a)
HOTAIR	Decrease	*Notch-1*	Inducing ovarian GC apoptosis	Zhao and Dong (2018)
DLEU1	Increase	miR-146b-5p	Promoting ovarian GC apoptosis	Zheng et al. (2021)
TRERNA1	Decrease	miR-23a	Promoting ovarian GC apoptosis	Zhang et al. (2022)
BBOX1-AS1	Increase	miR-146 b	Inducing ovarian GC apoptosis	Yu et al. (2022)
NORHA	Increase	miR-183–96–182	Promoting follicul aratresia	Yao et al. (2021)
Meg3	Increase	*P53/P66Shc*	Inhibiting mouse OGCs proliferation	Xiong et al. (2017)

## External factors involved in POF development and their epigenetic mechanisms

**FIGURE 3:**
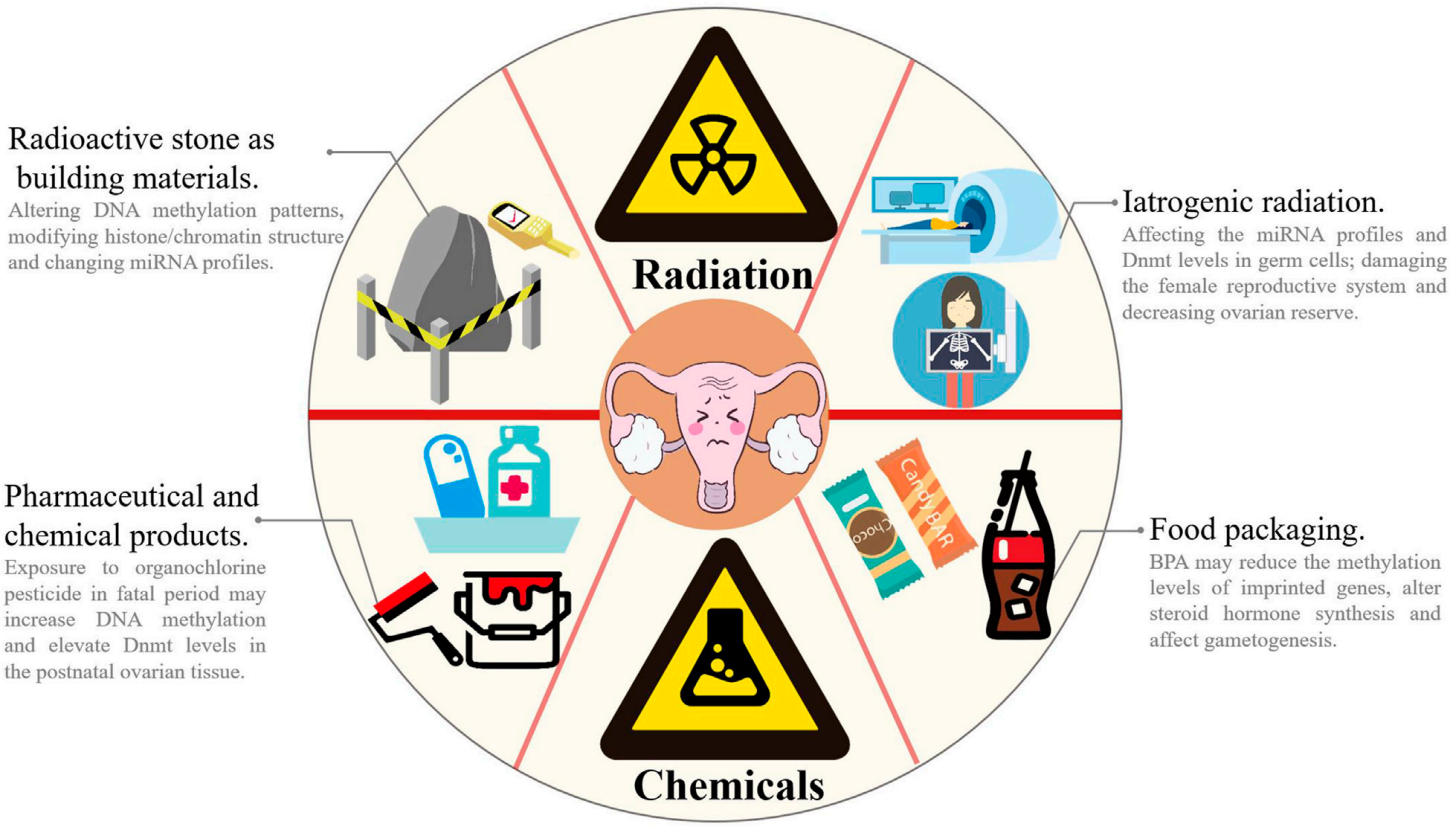
Environmental and lifestyle factors accelerates the decline of ovarian function. In our daily life, chemical and radiation pollutions are very common. Chemical pollutions come from paint (e.g., PCBs), drugs/pesticide (e.g., MXC and DDT), electronic equipment and food packaging (e.g., PAEs and BPA), and radiation pollutions mainly come from decoration materials (especially stones) and iatrogenic radiation (e.g., MRI and CT scan). Exposure to RTECs or ionizing radiation may alter DNA methylation patterns, modify histone/chromatin structure and change miRNA profiles and thus impairs sex hormone synthesis, affects gametogenesis and decreases fertility. MRI: magnetic resonance imaging; CT: computerized tomography.

Environmental, social, psychological and lifestyle factors may accelerate the decline in ovarian reserve (Richardson et al., 2014) (Figure 3). Based on accumulating evidence, exposure to reproductively toxic environmental chemicals (RTECs) leads to premature menopause and POF. RTEC exposure during fetal and neonatal periods reduces ovarian reserve, whereas exposure during the prepubertal period and adulthood accelerate follicular pool depletion, and the mechanism may be ascribed to changes in the germ cell epigenome (Béranger et al., 2012; Grindler et al., 2015; Vabre et al., 2017; Ge et al., 2019). According to a recent study, multiple chemicals (such as phthalates (PAEs), polychlorinated biphenyls (PCBs), and bisphenol A (BPA), which are widely used in electronic transformers, capacitors, coolants and food packaging), ionizing radiation, smoking and drinking may affect ovarian reserve *via* epigenetic regulation and induce POF (Yang et al., 2021). High-dose BPA exposure during the neonatal period is associated with the occurrence of POF in adulthood. Exposure to BPA during pregnancy has been suggested alter the expression of steroid hormone synthesis-related genes and microRNA profiles related to gonadal differentiation and follicle synthesis in offspring and ultimately disrupt the fertility of offspring (Yang et al., 2021). Zhang et al. found that exposure to BPA reduced the methylation levels of imprinted genes *insulin-like growth factor 2 receptor* (*Igf2r*), *paternally expressed gene 3* (*Peg3*) and *H19* in fetal rat germ cells, which in turn affected gametogenesis (Zhang et al., 2012). Qiu et al. reported decreased fertility and progesterone levels in neonatal mice exposed to BPA, along with increased serum testosterone and estradiol levels in their adult period. Exposure to BPA may interfere with the function of the hypothalamic-pituitary-ovarian axis (HPOA), decrease the production of sex hormones and reduce the number of oocytes (Qiu et al., 2020). Currently, researchers postulate that the reproductive toxicity of BPA is closely correlated with its epigenetic regulation of gene expression. Zama et al. found that when fetal rats were exposed to 100 mg/kg methoxychlor (MXC, an organochlorine pesticide with weak estrogenicity) *in utero* every day, the DNA methylation level in the estrogen receptor (ER)-β promoter region was increased, and the Dnmt3b level was elevated in postnatal rat ovarian tissue (Zama and Uzumcu 2009). Li et al. examined diethylhexyl phthalate (DEHP)-treated mouse oocytes and observed a reduced percentage of methylated CpG sites in the imprinted genes *Igfr2r* and *Peg3*, and the DNA methylation modification was inherited by the F2 generation and thus decreased the number of primordial follicles in puberty and adulthood (Li et al., 2014). Nilsson et al. reported that exposure to the environmental toxicants vinclozolin and dichlorodiphenyltrichloroethane (DDT) promotes epigenetic susceptibility in the ovaries of F0 generation rats, altering DNA methylation and ncRNAs in the ovarian GCs of the F3 generation; these results indicated that environmental toxicants may induce transgenerational inheritance of the ovarian GC epigenome (Nilsson et al., 2018). As shown in the study by Liu et al., a HFHS (high-fat and high-sugar) diet promotes ovarian GC aging and POF by inhibiting endogenous miR-146b-5p expression, activating the disabled homolog 2-interacting protein (DAB2ip)/apoptosis signal-regulating kinase 1 (ASK1)/p38 signaling pathway and γ-H2A.X phosphorylation (Liu et al., 2021c). Rhon-Calderón et al. found that daily exposure to low doses of 3-methylcholanthrene (3 MC) in adolescent mice decreases follicle numbers, blocks follicle development and inhibits ovulation (Rhon-Calderón et al., 2016). They indicated that 3 MC increases the H3K4me3 trimethylation levels at the *cytochrome P450 family one subfamily A member 1* (*Cyp1a1*), *jagged canonical notch ligand 1* (*Jag1*), *dnaJ heat shock protein family (Hsp40) member B6* (*Dnajb6*), *Igf2*, *Notch receptor 2* (*Notch2*), *ADAM with thrombospondin type 1 motif 1* (*Adamts1*), *BCL2-associated X* (*Bax*) and *Caspase-9* genes and the H3K9Ac acetylation levels at the *Cyp1A1*, *Jag1*, *cyclin-dependent kinase 2* (*Cdk2*), *Dnajb6*, *Igf2*, *intercellular cell adhesion molecule-1* (*Icam1*), and *Sp1* genes, suggesting that mechanisms may play an important role in POF development (Rhon-Calderón et al., 2018). Additionally, ionizing radiation alters DNA methylation patterns, modifies histone/chromatin structure and changes miRNA profiles (Pogribny et al., 2005; Halimi et al., 2012; Kumar et al., 2012; Metheetrairut and Slack 2013; Miousse et al., 2017). Ionizing radiation may also damage the female reproductive system and decrease ovarian reserve (Quan et al., 2020). Filkowski et al. found that exposure to 2.5 of Gy X-rays upregulated the expression level of miR-29 in mouse germ cells, resulting in a decrease in Dnmt3a levels, which in turn increased the susceptibility of the ovary in offspring and the risk of POF (Filkowski et al., 2010).

Transgenerational epigenetic changes may be inherited through germ cells (sperm or eggs) and occur in early embryonic and stem cells, affecting all somatic cells and tissues and increasing disease susceptibility in adulthood. Therefore, ovarian disease may be partially induced by ancestral environmental exposures and the corresponding epigenetic changes (Anway and Skinner 2008). With industrialization, globalization and the growing economy, people’s lifestyles have become more convenient than ever before. However, currently, people are exposed to various chemicals, and the potential hazards of these chemicals should not be ignored.

## Epigenetic targets OF POF therapy

Currently, the most common treatment for POF in the clinic is hormone replacement therapy (HRT). However, HRT only relieves low estrogen-related symptoms such as vaginal dryness, hot flashes and genitourinary tract atrophy but does not improve ovarian reproductive function (Sullivan et al., 2016). Therefore, an understanding of the pathogenesis of POF is crucial to develop corresponding treatment regimens targeting those key pathogenic factors. In many cases, POF patients harbor epigenetic alterations in their reproductive system instead of genetic alterations (e.g., gene mutations). Therefore, precision therapy targeting epigenetic variations is a probable and valuable approach for POF clinical treatment in the future.

Epigenetic strategies for POF treatment targeting DNA and histone modifications are still in the exploratory stage. Previous studies have confirmed that butyrate, an HDAC inhibitor, can loosen the chromosome structure and enhance gene transcription by increasing the level of histone acetylation (Corfe 2012). Ye et al. found that butyrate increases estradiol and progesterone synthesis in rat and human GCs by increasing the acetylation of histone H3K9 (H3K9ac) and stimulating the peroxisome proliferator activated receptor (PPARγ)/CD36/steroidogenic acute regulatory protein (StAR) pathways, which enhances mitochondrial dynamics and alleviates oxidative damage in GCs (Ye et al., 2021). As shown in the study by Liu et al., thymopentin promotes the transcription and expression of Lin28A (a marker of ovarian GC proliferation), inhibits the activity of let-7 family miRNAs and alleviates the aging of ovarian GCs, which provides a valuable therapeutic target for POF (Liu et al., 2021b). Zhao and others found that hyaluronic acid (HA) blocks Tripterygium glycoside-induced POI-like presentations in rats, including delayed or irregular estrous cycles, reduced E2 concentrations, decreased numbers of follicles, destruction of the follicle structure, and damage to the reproductive ability. Regarding the molecular mechanism, they indicated that HA upregulated progesterone receptor membrane component 1 (Pgrmc 1) expression in GCs by suppressing of miR-139-5p. Moreover, HA downregulated miR-139-5p expression *via* histone deacetylation at its promoter (Zhao et al., 2014; Zhao et al., 2015).

Natural products and traditional Chinese medicine may exert unexpected effects on the treatment of POF. For example, Zhu et al. showed that American ginseng treatment regulates the expression levels of phospholipase A2 group IVA (Pla2g4a), miR-29a and miR-144 in POF rats. After POF rats were administered American ginseng for 1 month, the levels of all hormones (prostaglandin E2 (PGE2), FSH, and luteinizing hormone (LH)) and E2 secretion approached normal levels, and POF symptoms were improved (Zhu et al., 2015). Liu et al. treated POF rats with 10 ml/kg/day modified Bazhen decoction (MBD, a traditional Chinese medicine mixing *Ginseng*, *Atractylodes macrocephala Koidz*, *Poria cocos*, *Licorice*, *Angelica sinensis*, *Radix Rehmanniae*, *Radix Paeoniae Alba* and *Ligusticum wallichii*) for 4 weeks and found that MDB substantially activated X-linked inhibitor of apoptosis protein (Xiap) but inhibited the expression of miR-23a and miR-27a and effectively prevented the apoptosis of oocytes and GCs (Liu et al., 2019a).

Mesenchymal stem cell (MSC) transplantation is the most promising option to treat POF. Preliminary clinical trials have shown that MSCs improve ovarian function and increase the pregnancy rate of POI/POF patients (Wang et al., 2021b). We systematically described the mechanism and application of MSC therapy for POF in our previous review (Wang et al., 2021b). By synthesizing the current research conclusions, we consider that MSC therapy for POF relies mainly on miRNA-dependent epigenetic regulation and its related cellular signal transduction pathways. According to Fu et al., transplantation of MSCs overexpressing miR-21 in rats with chemotherapy-induced POF increases ovarian weight and follicle counts, increases E2 levels and decreases FSH levels. They also indicated that miR-21 inhibits GC apoptosis by targeting phosphatase and tensin homolog (Pten) and programmed cell death 4 (Pdcd4) (Fu et al., 2017). EL-Derany et al. showed that bone marrow MSCs (BMMSCs) increase the ovarian follicle pool and preserve ovarian function in rats with γ-ray-induced POF. They indicated that BMMSC miRNAs epigenetically regulate the TGF-β, Wnt/β-catenin and Hippo signaling pathways, which control the apoptosis, proliferation, and differentiation of ovarian follicles (El-Derany et al., 2021).

Exosomes and their incorporated miRNAs have been proven to be functional components in MSC therapy. Yang et al. found that BMSC-derived exosomes prevent ovarian follicular atresia and inhibit GC apoptosis in cyclophosphamide-treated rats *via* the delivery of miR-144-5p and suppression of its related Pten/Pi3k/Akt pathway (Yang et al., 2020). Sun et al. found that miR-644-5p carried by BMSC-derived exosomes represses GC apoptosis and increase cell viability in mice with cisplatin-induced POF by negatively regulating p53 (Sun et al., 2019). In the study by Xiao et al., amniotic fluid stem cell (AFSC)-derived exosomes prevented ovarian follicular atresia in chemotherapy-treated mice *via* the delivery of miR-146a (targeting *Irak1* and *Traf6*) and miR-10a (targeting *Bim*) and inhibition of GC apoptosis (Xiao et al., 2016).

Currently, hundreds of potential epigenetic drugs targeting DNMTs and histone methyltransferase have been developed or have undergone clinical trials, and some of them have been approved by the FDA or EMA. However, the diseases for which these drugs have been approved as treatments are limited to cancers, Alzheimer’s disease, and diabetes (Pasyukova et al., 2021), and no epigenetic drug is definitely suitable for the prevention or treatment of POF (Table 6). Additionally, since aging is highly complex, the side effects of some epigenetic drugs have been introduced into clinical practice to treat aging-associated diseases (e.g., neurological disturbances, and cardiac and metabolic abnormalities) are unignorable (Nervi et al., 2015).

**TABLE 6:** Epigenetic targets of POF therapy.

Treatment reagents or methods	Preclinical/Clinical	Molecular mechanism	Treatment effect	References
Butyric acid	Preclinical	Upregulating histone H3K9 acetylation *via* PPARγ and PGC1α pathways	Promoting synthesis of estradiol and progesterone in ovarian GCs	Ye et al. (2021)
Thymopentin	Preclinical	Promoting the transcriptional activation of Lin28a by stimulating the expression of transcription factor YY2 and inhibiting the activity of LET-7 family miRNAs	Inhibiting ovarian GC apoptosis	Liu et al. (2021b)
Hyaluronic Acid	Preclinical	Downregulating miR-139-5p levels by histone acetylation and enhancing the expression level of PGRMC1 in GC	Inhibiting ovarian GC apoptosis	Zhao et al. (2014)
Changing eating habits	Preclinical	Upregulating endogenous miR-146b-5p expression, inhibiting DAB2ip/ask1/p38 signaling pathway and γ-H2A.X phosphorylation modification	Inhibiting ovarian GC aging	Liu et al. (2021c)
American Ginseng	Preclinical	Downregulating PLA2G4A mRNA and protein expression; Increasing miR-29a and miR-144 levels; Decreasing serum prostaglandin PGE2, LH and FSH levels; Increasing E2 level	Promoting prostaglandin biosynthesis and reproductive hormone synthesis and ovulation	Zhu et al. (2015)
Flavored Bazhen Soup	Preclinical	Inhibiting the expression levels of miR-23a and miR-27a; Activating XIAP	Preventing oocytes and GCs apoptosis	Liu et al. (2019a)
BMSCs	Preclinical	Promoting miR-21 overexpression and downregulating PTEN and PDCD4 levels	Inhibiting ovarian GC apoptosis	Fu et al. (2017)
	Preclinical	Upregulating Wnt/β-catenin and HIPPO signaling pathways	Promoting follicle growth and maturation	El-Derany et al. (2021)
	Preclinical	The derived exosomal miR-144-5p may activate the PTEN/PI3K/AKT pathway	Inhibiting follicular atresia	Yang et al. (2020)
	Preclinical	miR-644-5p carried by exosomes can target and downregulate p53 level	Inhibiting ovarian GC apoptosis	Sun et al. (2019)
AFSC	Preclinical	miR-146a carried by exosomes downregulates the expression of target genes *IRAK1* and *Traf632*	Inhibiting ovarian GC apoptosis	Xiao et al. (2016)
	Preclinical	miR-10a carried by exosomes can inhibit the proapoptotic factor Bim31	Reducing ovarian GC apoptosis	Xiao et al. (2016)

## Conclusion and prospects

POF is different from aging-associated diseases, such as Alzheimer’s disease, diabetes and cancers. Although non-fatal, it is very harmful to women’s bodies and minds, especially those women of childbearing age. In China, with the continuous development of the economy and revolution of traditional culture, women are increasingly participating in all aspects of social life. Due to increasing academic, economic and living pressure, the maternal age at first pregnancy of Chinese women continues to increase. If this phenomenon continues, it will inevitably lead to an aging population and a substantial decrease in the social labor force. Moreover, many women find themselves suffering from POF by the time they want to have a child, and an indisputable fact is that the number of POF patients is increasing in the clinic.

Due to the non-lethal nature of POF, many women choose to remain silent about the disease. The complexity of the disease prevents researchers and doctors from determining a clear and definite cause of the disease. However, the spread of this disease will clearly harm society in the future. In many cases, the occurrence of POF is not caused by an exact gene or protein variation; it is more likely induced by the combined effect of the patient’s physical condition, psychological condition and living environment. In this case, patients’ epigenetic changes are the dominant factor contributing to this disease. In this review, by summarizing the research achievements published in the past 2 decades, we draw several conclusions, which are listed below. 1) Epigenetic variation exists objectively in POF patients and is closely correlated with the occurrence and development of POF. 2) Epigenetic variations in POF patients mainly include changes in DNA, histones, enzymes, and profiles of ncRNAs. 3) Studies examining ncRNA expression profiles are significantly more common than studies on assessing other aspects, which may be because next-generation sequencing is easier, faster and cheaper to implement. Meanwhile, we strongly recommend that the following issues are considered: 1) research on the epigenetic etiology of POF is still far from sufficient; 2) although closely related to epigenetic variations, no epigenetic drug specific for POF has been developed. In other words, the treatment of this disease is still not receiving sufficient attention; 3) as a chronic disease, stem cell therapy and natural medicine (mainly targeting non-coding RNA and immunomodulation) appear to be milder and more appropriate than epigenetic enzyme inhibitors.
